# Evaluation of filaggrin 2 expression in dogs with atopic dermatitis before and after oclacitinib maleate administration

**DOI:** 10.1111/vde.13334

**Published:** 2025-03-05

**Authors:** Wendie Roldan Villalobos, Tássia Ferreira, Fernanda Borek, Domenico Santoro, Lluis Ferrer, Marconi Farias

**Affiliations:** ^1^ Department of Veterinary Medicine, School of Medicine and Life Sciences Pontifical Catholic University of Paraná Curitiba PR Brazil; ^2^ Department of Small Animal Clinical Sciences, College of Veterinary Medicine University of Florida Gainesville Florida USA; ^3^ Department of Animal Medicine and Surgery Universitat Autònoma de Barcelona (UAB) Barcelona Spain

**Keywords:** atopic dermatitis, dog, filaggrin, oclacitinib, skin barrier

## Abstract

**Background:**

Canine atopic dermatitis (cAD) is a chronic, inflammatory, multifactorial and pruritic disease. The presence of skin barrier impairment (e.g. filaggrin alterations), along with abnormal immune responses, can negatively impact cutaneous barrier function.

**Objectives:**

To evaluate the filaggrin 2 (FLG2) expression in atopic dogs before and after the administration of oclacitinib maleate.

**Animals:**

Sixteen privately owned dogs with a diagnosis of cAD and 10 healthy control dogs.

**Materials and Methods:**

Oclacitinib maleate monotherapy at 0.5 mg/kg, orally, twice‐daily for the first 14 days and once‐daily for 16 additional days, was administered to the atopic dogs. Skin biopsies from lesional and nonlesional skin were obtained from atopic dogs on Day(D)0 and D30 and from the same anatomical locations from the control group on D0. Immunohistochemical investigation was performed using a primary custom‐made anti‐canine‐filaggrin 2 polyclonal antibody. Immunolabelled slides were scanned and FLG2 expression was measured. Data were analysed and a *p*‐value ≤0.05 was considered statistically significant.

**Results:**

There was a higher FLG2 expression in control skin when compared with atopic skin (lesional and nonlesional) on D0 (*p* = 0.033). FLG2 expression comparison between control and D30 (nonlesional) did not show a significant difference (*p* = 0.509). A significant increase in FLG2 expression in atopic nonlesional skin on D30 compared with nonlesional skin on D0 was also observed (*p* = 0.014).

**Conclusions and Clinical Relevance:**

Oclacitinib maleate could have a positive impact on cutaneous barrier structure, improving FLG2 expression by decreasing inflammation and cutaneous trauma.

## Graphical abstract

**Background –** Canine atopic dermatitis (cAD) is a chronic, inflammatory, multifactorial and pruritic disease. The presence of skin barrier impairment (e.g. filaggrin alterations), along with abnormal immune responses, can negatively impact cutaneous barrier function. **Objectives –** To evaluate the filaggrin 2 (FLG2) expression in atopic dogs before and after the administration of oclacitinib maleate. **Conclusions and Clinical Relevance –** Oclacitinib maleate could have a positive impact on cutaneous barrier structure, improving FLG2 expression by decreasing inflammation and cutaneous trauma.

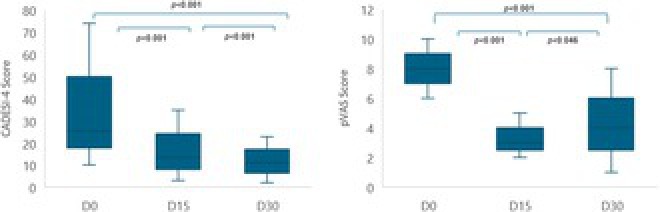

## Zusammenfassung

**Hintergrund:**

Die atopische Dermatitis des Hundes (cAD) ist eine chronische, entzündliche, multifaktorielle und juckende Erkrankung. Das Auftreten einer Beeinträchtigung der Hautbarriere (z.B. Filaggrinveränderungen), zusammen mit abnormalen Immunantworten, kann sich negativ auf die Funktion der Hautbarriere auswirken.

**Ziele:**

Eine Evaluierung der Filaggrin 2 (FLG2) Exprimierung bei atopischen Hunden vor und nach der Verabreichung von Oclacitinib Maleat.

**Tiere:**

Sechzehn Hunde in Privatbesitz mit der Diagnose einer cAD und 10 gesunde Kontrollhunde.

**Materialien und Methoden:**

Es wurde den atopischen Hunden eine Monotherapie mit Oclacitinib Maleat bei einer Dosis von 0,5 mg/kg *per os* zweimal täglich für die ersten 14 Tage und einmal täglich für weitere 16 Tage verabreicht. Es wurden Hautbiopsien von läsionaler und nicht läsionaler Haut von den atopischen Hunden am Tag (D)0 und D30 und bei der Kontrollgruppe von denselben anatomischen Stellen am D0 genommen. Es wurde eine immunhistochemische Untersuchung mittels primärem speziell‐angefertigtem Anti‐canine‐Filaggrin 2 polyklonalem Antikörper durchgeführt. Es wurden die immunhistochemisch gefärbten Objektträger gescannt und FLG2 Exprimierung gemessen. Die Daten wurden analysiert und ein *p*‐Wert ≤0,05 wurde als statistisch signifikant erachtet.

**Ergebnisse:**

Es bestand eine höhere FLG2 Exprimierung in der Kontrollhaut im Vergleich zur atopischen Haut (läsional und nicht‐läsional) am D0 (*p* = 0,033). Der Vergleich der FLG2 Exprimierung zwischen den Kontrollen und D30 (nicht‐läsional) zeigte keine signifikanten Unterschiede (*p* = 0,509). Eine signifikante Erhöhung der FLG2 Exprimierung in atopischer nicht‐läsionaler Haut am D30 im Vergleich zu nicht‐läsionaler Haut am D0 konnte ebenso beobachtet werden (*p* = 0,014).

**Schlussfolgerungen und klinische Bedeutung:**

Oclacitinib Maleat könnte eine positive Auswirkung auf die Struktur der Hautbarriere haben, da die FLG2 Exprimierung durch Abnahme der Entzündung und des kutanen Traumas verbessert wird.

## 摘要

**背景:**

犬特应性皮炎 (cAD) 是一种慢性、炎性、多因素和瘙痒性疾病。皮肤屏障受损(例如丝聚蛋白改变)以及异常免疫反应会对皮肤屏障功能产生负面影响。

**目标:**

评估在给予马来酸奥拉替尼之前和之后特应性犬的丝聚蛋白 2 (FLG2) 表达。

**动物:**

16 只被诊断为 cAD 的私家犬和 10 只健康对照犬。

**材料和方法:**

给特应性犬服用马来酸奥拉替尼单药治疗，前 14 天每天两次口服 0.5 mg/kg，之后 16 天每天一次。在第 0 天和第 30 天从特应性犬的病变和非病变皮肤获取皮肤活检样本，并在第 0 天从对照组的相同解剖位置获取皮肤活检样本。使用定制的抗犬丝聚蛋白 2 多克隆抗体进行免疫组织化学研究。扫描免疫标记的载玻片并测量 FLG2 表达。分析数据，*p* 值≤ 0.05 被认为具有统计学意义。

**结果:**

与 D0 上的特应性皮肤(病变和非病变)相比，对照皮肤中的 FLG2 表达更高(*p* = 0.033)。对照和 D30(非病变)之间的 FLG2 表达比较没有显示出显着差异(*p* = 0.509)。与 D0 上的非病变皮肤相比，D30 上的特应性非病变皮肤中的 FLG2 表达也显著增加(*p* = 0.014)。

**结论和临床相关性:**

马来酸奥拉替尼可以对皮肤屏障结构产生积极影响，通过减少炎症和皮肤创伤来改善 FLG2 表达。

## Résumé

**Contexte:**

La dermatite atopique canine (DAC) est une maladie chronique, inflammatoire, multifactorielle et prurigineuse. La présence d'une altération de la barrière cutanée (par exemple, des altérations de la filaggrine), ainsi que des réponses immunitaires anormales, peuvent avoir un impact négatif sur la fonction de barrière cutanée.

**Objectifs:**

Évaluer l'expression de la filaggrine 2 (FLG2) chez des chiens atopiques avant et après l'administration de maléate d'oclacitinib.

**Animaux:**

Seize chiens de propriétaires avec un diagnostic de DAC et 10 chiens témoins en bonne santé.

**Matériels et méthodes:**

Les chiens atopiques ont reçu du maléate d'oclacitinib en monothérapie à raison de 0,5 mg/kg par voie orale, deux fois par jour pendant les 14 premiers jours et une fois par jour pendant 16 jours supplémentaires. Des biopsies cutanées lésionnelles et non lésionnelles ont été prélevées sur des chiens atopiques à J0 et J30 et sur les mêmes sites anatomiques du groupe témoin à J0. L'étude immunohistochimique a été réalisée à l'aide d'un anticorps polyclonal primaire anti‐canine‐filaggrine 2 fabriqué sur mesure. Les lames immunomarquées ont été scannées et l'expression de FLG2 a été mesurée. Les données ont été analysées et une valeur *p* ≤ 0,05 a été considérée comme statistiquement significative.

**Résultats:**

L'expression du FLG2 était plus élevée dans la peau témoin que dans la peau atopique (lésionnelle et non lésionnelle) à J0 (*p* = 0,033). La comparaison de l'expression du FLG2 entre la peau témoin et la peau atopique (lésionnelle et non lésionnelle) à J30 n'a pas montré de différence significative (*p* = 0,509). Une augmentation significative de l'expression du FLG2 dans la peau atopique non lésionnelle à J30 par rapport à la peau non lésionnelle à J0 a également été observée (*p* = 0,014).

**Conclusions et pertinence clinique:**

Le maléate d'oclacitinib pourrait avoir un impact positif sur la structure de la barrière cutanée, améliorant l'expression du FLG2 en diminuant l'inflammation et les traumatismes cutanés.

## 要約

**背景:**

犬アトピー性皮膚炎(cAD)は、慢性、炎症性、多因子性、掻痒性疾患である。皮膚バリア障害(例:フィラグリン変化)の存在は、異常な免疫反応とともに皮膚バリア機能に悪影響を及ぼす可能性がある。

**目的:**

本研究の目的は、オクラシチニブマレイン酸塩投与前後のアトピー犬におけるフィラグリン2(FLG2)の発現を評価することであった。

**対象動物:**

cADと診断された16頭のオーナー所有犬および10頭の健常対照犬。

**材料と方法:**

オクラシチニブマレイン酸塩単剤療法0.5mg/kgを、最初の14日間は1日2回、追加の16日間は1日1回経口投与した。試験開始0日目および30日目にアトピー犬の病変部および非病変部のから、試験開始0日目に対照群の同じ解剖学的部位から皮膚生検を得た。免疫組織化学的検査は、一次カスタムメイド抗犬フィラグリン2ポリクローナル抗体を用いて行った。免疫標存したスライドをスキャンし、FLG2の発現を測定した。データを分析し、p値≦0.05を統計的に有意とみなした。

**結果:**

試験開始0日目のアトピー群の皮膚(病変部および非病変部)と比較すると、対照群の皮膚ではFLG2発現が高かった(*p* = 0.033)。試験開始30日目(非病変性)のFLG2発現の比較では、対照と有意差は認められなかった(*p* = 0.509)。試験開始30日目におけるアトピー群の非病変部皮膚では、FLG2発現が試験開始0日目と比較して有意に増加した(*p* = 0.014)。

**結論と臨床的意義:**

オクラシチニブマレイン酸塩は、炎症および皮膚外傷を軽減することによりFLG2発現を改善し、皮膚バリア構造に好影響を与える可能性があった。

## Resumo

**Contexto:**

A dermatite atópica canina (DAC) é uma doença crônica, inflamatória, multifatorial e pruriginosa. A presença de comprometimento da barreira cutânea (ex: alterações da filagrina), juntamente com respostas imunológicas anormais, pode impactar negativamente a função desta barreira.

**Objetivos:**

Avaliar a expressão da filagrina 2 (FLG2) em cães atópicos antes e depois da administração de maleato de oclacitinib.

**Animais:**

Dezesseis cães de propriedade privada com diagnóstico de DAC e 10 cães controle saudáveis.

**Materiais e métodos:**

A monoterapia com maleato de oclacitinib a 0,5 mg/kg, por via oral, duas vezes ao dia durante os primeiros 14 dias e uma vez ao dia durante 16 dias adicionais, foi administrada aos cães atópicos. Biópsias de pele de pele lesional e não lesional foram obtidas de cães atópicos no Dia (D)0 e D30 e das mesmas localizações anatômicas do grupo controle no D0. A investigação imuno‐histoquímica foi realizada utilizando um anticorpo policlonal primário personalizado anti‐filaggrina 2 canina. As lâminas imunomarcadas foram escaneadas e a expressão de FLG2 foi medida. Os dados foram analisados e um valor de *p* ≤ 0,05 foi considerado estatisticamente significativo.

**Resultados:**

Houve uma expressão de FLG2 maior na pele controle quando comparada com a pele atópica (lesional e não lesional) no D0 (*p* = 0,033). A comparação da expressão de FLG2 entre controle e D30 (não lesional) não mostrou uma diferença significativa (*p* = 0,509). Um aumento significativo na expressão de FLG2 na pele atópica não lesional no D30 em comparação com a pele não lesional no D0 também foi observado (*p* = 0,014).

**Conclusões e relevância clínica:**

O maleato de oclacitinib pode ter tido um impacto positivo na estrutura da barreira cutânea, melhorando a expressão de FLG2 ao diminuir a inflamação e o trauma cutâneo.

## RESUMEN

**Introducción:**

La dermatitis atópica canina (cAD) es una enfermedad crónica, inflamatoria, multifactorial y pruriginosa. La presencia de alteraciones de la barrera cutánea (p. ej., alteraciones de la filagrina), junto con respuestas inmunitarias anormales, puede afectar negativamente la función de la barrera cutánea.

**Objetivos:**

Evaluar la expresión de la filagrina 2 (FLG2) en perros atópicos antes y después de la administración de maleato de oclacitinib.

**Animales:**

Dieciséis perros de propietarios privados con diagnóstico de cAD y 10 perros de control sanos.

**Materiales y métodos:**

Se administró a los perros atópicos una monoterapia con oclacitinib maleato a 0,5 mg/kg, por vía oral, dos veces al día durante los primeros 14 días y una vez al día durante 16 días adicionales. Se obtuvieron biopsias de piel de perros atópicos con y sin lesiones el día (D)0 y el día 30 y de las mismas ubicaciones anatómicas del grupo de control el día 0. Se realizó una investigación inmunohistoquímica utilizando un anticuerpo policlonal anti‐filagrina canina 2 personalizado. Se escanearon portaobjetos inmunomarcados y se midió la expresión de FLG2. Se analizaron los datos y se consideró estadísticamente significativo un valor *p* ≤ 0,05.

**Resultados:**

Hubo una mayor expresión de FLG2 en la piel de control en comparación con la piel atópica (lesional y no lesional) en D0 (*p* = 0,033). La comparación de la expresión de FLG2 entre el control y D30 (no lesional) no mostró una diferencia significativa (*p* = 0,509). También se observó un aumento significativo en la expresión de FLG2 en la piel atópica no lesional en D30 en comparación con la piel no lesional en D0 (*p* = 0,014).

**Conclusiones y relevancia clínica:**

El maleato de oclacitinib podría tener un impacto positivo en la estructura de la barrera cutánea, mejorando la expresión de FLG2 al disminuir la inflamación y el trauma cutáneo.

## INTRODUCTION

Canine atopic dermatitis (cAD) results from the interaction between genetics and environmental factors that shape the immune response and skin barrier function.^1^ Epidermal barrier dysfunction occurs in both human AD and canine (c)AD, allowing penetration of irritants, micro‐organisms, microbial antigens and environmental allergens. This stimulates the local immune system and induces inflammatory responses.^2^ Aberrant lipid composition, defective and decreased structural proteins, increased skin pH and reduced skin microbiome diversity are some of the known skin barrier alterations present in human patients with AD.^3^

Filaggrin (FLG) belongs to the S100 fused‐type protein family along with filaggrin 2 (FLG2), trichohyalin and hornerin, among others, and plays a critical role in epidermal differentiation and cutaneous barrier function.^4^ It derives from profilaggrin, a precursor formed by several FLG units and stored in keratohyalin granules of the stratum granulosum.^4^ Breakdown of FLG generates natural moisturising factors that maintain hydration and lower pH, and also contribute to skin antimicrobial protection. FLG deficiency leads to increased epidermal pH, which stimulates serine protease activity to degrade corneodesmosomes and inhibit ceramides production.^3^

Mutations affecting the C‐terminal portion of the *FLG* gene are one of the best confirmed risk factors for the development of AD in humans.^5^

In dogs with AD, the disruption of the skin barrier has been hypothesised. Decreased immunohistochemical staining,^5^, ^6^ mRNA altered expression, decreased^7^ or increased^8^ and nonhomogeneous distribution of FLG have been described as potential factors involved in the pathogenesis of cAD.^1^ In dogs and humans, FLG and FLG2 have similar locations within the epidermis.^9^ In humans, FLG2 is expressed in the stratum granulosum where it is processed into smaller fragments by the protease calpain 1.^10^ The amino terminal domain of FLG2 is a component of cornified envelopes and co‐localises with corneodesmosin, implying that FLG2 participates in epidermal and stratum corneum adhesion. FLG2 alterations in the skin of atopic humans^10^ and dogs^11^, ^12^ have been described.

Abnormal immune responses also have been linked to the pathogenesis of AD. In humans, it is documented that T‐helper (Th)2, Th22 and Th17 cells are involved in the acute stage of the disease.^11^, ^13^ Th2‐polarised immune responses have been demonstrated in cAD with increased levels of interleukin (IL)‐4, IL‐5 and IL‐13 in the serum, peripheral blood mononuclear cells and lesional skin.^14^ IL‐31 also is a key mediator of pruritus in cAD,^15^ by activating skin‐innervating sensory neurons, as well as by enhancing the release of pro‐inflammatory mediators from keratinocytes and immune cells.^15^

It is remarkable that, in addition to *FLG* gene mutations, type 2 inflammatory mediators, as well as inflammation mediated by Th17, Th22 and Th1, can also reduce FLG expression, as reported in humans with AD.^16^

Oclacitinib maleate is a janus kinase (JAK) inhibitor labelled for use in dogs for the control of pruritus associated with allergic dermatitis. It is effective at inhibiting JAK1, which plays a pivotal role in mediating the intracellular signalling of IL‐2, IL‐4, IL‐6, IL‐13 and IL‐31, cytokines involved in allergy, inflammation and pruritus.^17^

Considering the relevance of Th2 responses in the pathogenesis of cAD and their possible involvement in cutaneous barrier alterations, we hypothesised that FLG2 expression in dogs with AD improves after the use of oclacitinib maleate. Hence, the aim of this study was to evaluate the FLG2 expression in the epidermis of atopic dogs before and after the administration of oclacitinib maleate.

## MATERIALS AND METHODS

### Institutional protocol review and approvals

This study was approved by the Ethics Committee on Animal Use (CEUA) of the Pontifical Catholic University of Paraná PUC‐PR (No. 2248). Informed client consent was obtained by signature at the time of enrolment of each dog in both groups.

### Animals

Sixteen privately owned dogs with a diagnosis of AD, comprising seven mixed‐breed, five shih‐tzus, two Lhasa‐apso, one beagle and one golden retriever (age range 1.5–12 years) and 10 healthy control dogs, with no history or clinical signs of skin disease, comprising seven mixed‐breed, two Yorkshire terrier and one pug (age range 1–12 years) were included in this study.

Diagnosis of AD was based on compatible history, the presence of five or more signs under Favrot's 2010 criteria^18^ and the exclusion of other causes of pruritus, through skin/ear canal cytological investigation and skin scrapings to rule out concurrent infections/infestations. Before enrolment, if present, secondary bacterial and/or yeast infections were addressed by appropriate antimicrobial therapy (topical/systemic medication). An ectoparasiticide (Afoxolaner Nexgard: Boehringer Ingelheim) was administered orally to the atopic dogs, also, according to body weight. No anti‐inflammatory medications were given for ≥4 weeks before inclusion. Oclacitinib maleate (Apoquel: Zoetis) at 0.5 mg/kg, orally, twice‐daily for the first 14 days and once‐daily for 16 additional days, was administered to the atopic dogs. Concurrent topical therapies (i.e. antiseptics and moisturisers) were not allowed during the study period.

Dogs aged <12 months, weighing <3 kg, with nervous and/or aggressive behaviour, history or evidence of neoplasia, demodicosis and/or severe infections, as well as dogs exhibiting chronic AD (e.g. lichenification) and pregnant/lactating females, were excluded from the study.

### Clinical evaluation

Canine Atopic Dermatitis Extent and Severity Index—4th iteration (CADESI‐04)^19^ and pruritus Visual Analog Scale (pVAS)^20^ evaluations were performed in the atopic dogs on Day (D)0, D15 and D30.

### Skin biopsies

Punch skin biopsies (6 mm diameter) were taken from lesional (erythema—axillae/inguinal area) and nonlesional skin (inguinal area) from the atopic dogs on D0 (before oclacitinib maleate) and D30 (after oclacitinib maleate), and from the same anatomical locations from the control group on D0. Biopsies were obtained after local anaesthesia with 2% lidocaine and were sutured routinely.

### Assessment of cutaneous filaggrin expression by immunohistochemical investigation

#### Processing of samples

Skin biopsies from atopic dogs (lesional and nonlesional) obtained on D0 and D30 and from control dogs obtained on D0 were fixed in 10% neutral buffered formalin for 48 h and then subjected to standard histological processing. Tissue microarrays were prepared to perform immunohistochemical investigation. Slides were deparaffinised and subjected to antigen retrieval at pH 9.0. Then, they were quenched in 3% hydrogen peroxide, and a primary custom‐made anti‐canine‐filaggrin 2 polyclonal antibody (University of Florida, USA),^11^, ^21^, ^22^ diluted at 1:500, was added for 1.5 h. Histofine 3,3´‐diaminobenzoide substrate (Nichirei Biosciences) was used for the visualisation of the immunolabelling, and slides were counterstained with haematoxylin. Between reactions, slides were washed in distilled water and phosphate buffered saline.

#### Imaging and image analysis

Immunolabelled slides were scanned (Axio Scan.Z1; Zeiss) obtaining 20 images for each animal using the software ZEN blue edition (Zeiss). Images were obtained from random areas and analysed blindly, with no interference from the observer. FLG immunolabelling scans were measured through the software image pro‐plus v4.5 (Media Cybernetics), using a colour semiautomated segmentation method, which artificially delimited and quantified the area of tissue immunoexpression. Immunolabelling values were initially obtained in absolute areas (μm^2^) and then converted into percentages through the formula: % = (immunopositive area/ total tissue area) × 100. An immunolabelling average for each animal was calculated and analysed statistically.

### Statistics

Normality of data distribution was tested using the Shapiro–Wilk test. Student's *t‐*test and ANOVA for independent groups and *t‐*test for paired samples were used for parametric variables. Wilcoxon–Mann–Whitney and Kruskal–Wallis tests for independent groups as well as Wilcoxon and Friedman tests for dependent groups were used for nonparametric variables. Spearman coefficient was used to correlate two continuous nonparametric variables. A *p*‐value ≤0.05 was considered significant. For data analysis, JMP pro 14.0.0 (SAS Institute) was used.

## RESULTS

**FIGURE 1:**
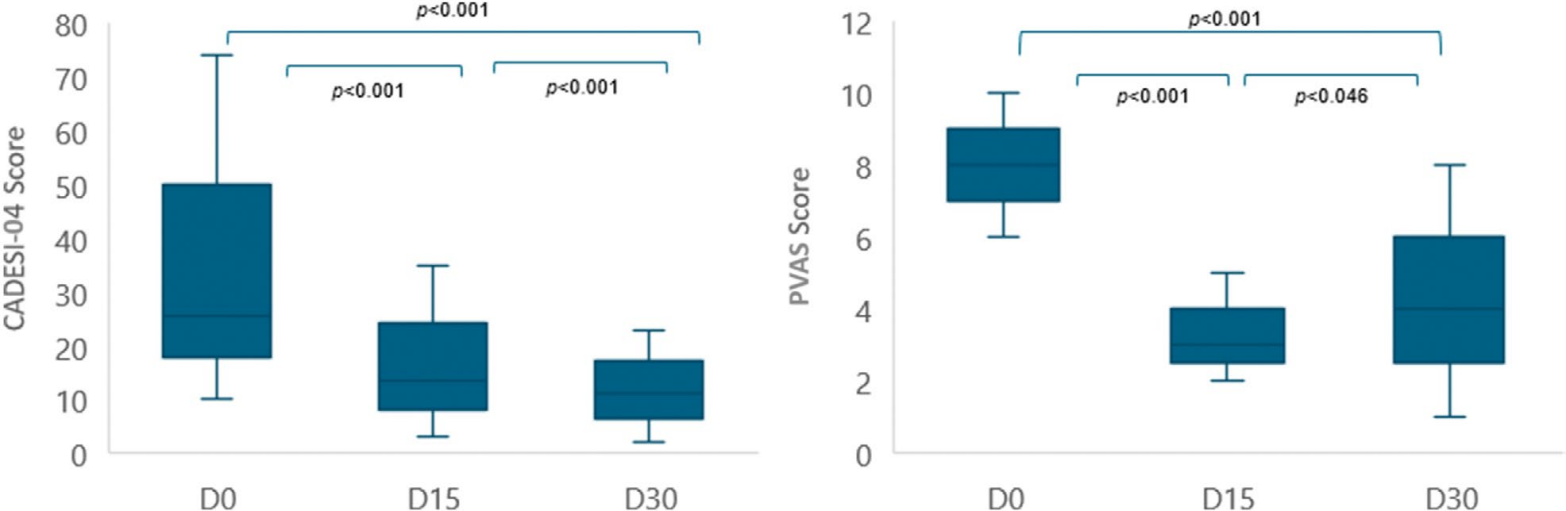
Reduction of the clinical scores Canine Atopic Dermatitis Extent and Severity Index—4th iteration (CADESI‐04) and pruritus Visual Analog Scale (pVAS) associated with the use of oclacitinib maleate.

A significant reduction in the clinical scores, CADESI‐04 (*p* < 0.001 between D0, D15 and D30) and PVAS (*p* < 0.001 for D15 and D30 compared with D0 and *p* = 0.046 between D15 and D30), associated with the use of oclacitinib maleate was observed (Figure 1).

**FIGURE 2:**
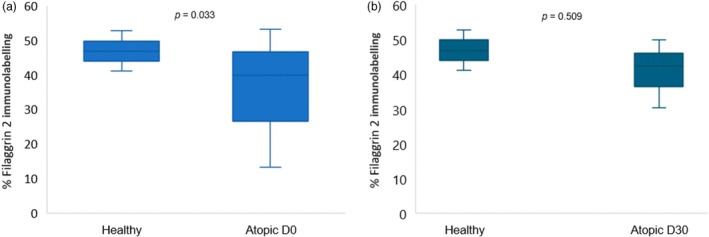
Comparisons of filaggrin 2 expression between control and atopic skin (lesional and nonlesional) on Day (D)0 (a), and between control and atopic skin on D30 (nonlesional) after oclacitinib maleate (b).

**FIGURE 3:**
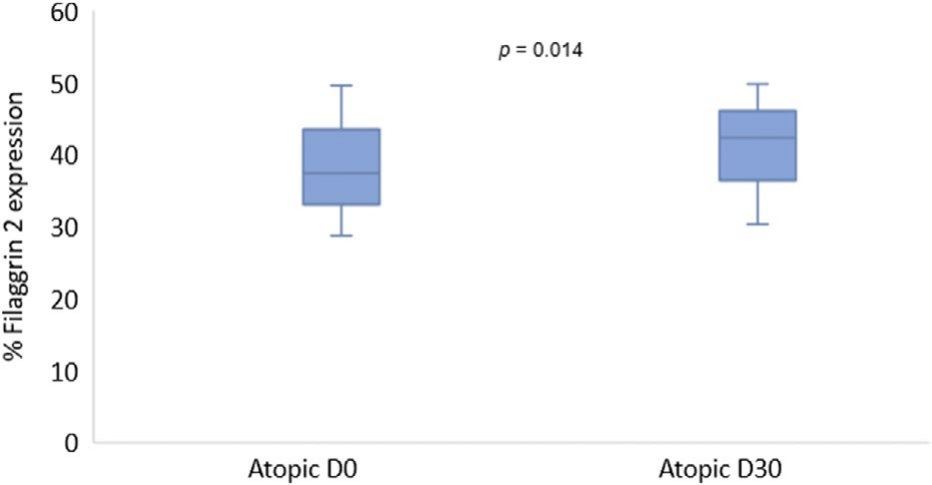
Comparison of filaggrin 2 expression in nonlesional atopic skin on Day (D)0 (before oclacitinib maleate) and D30 (after oclacitinib maleate).

For the percentage of FLG2 immunostaining, there was a higher FLG2 expression in control skin when compared with atopic skin (lesional and nonlesional) on D0 (*p* = 0.033) (Figure 2a). Interestingly, the comparison of FLG2 expression between control and D30 (nonlesional) did not show a significant difference (*p* = 0.509) (Figure 2b). A significant increase in FLG2 expression in atopic skin on D30 compared with nonlesional on D0 (*p* = 0.014) (Figure 3) also observed. Conversely, the comparison between FLG2 immunolabelling of lesional skin on D0 and D30 did not exhibit a significant difference (*p* = 0.688) (see Figure S1).

Spearman's matrix showed no positive correlation between FLG2 expression and clinical scores, CADESI‐04 (D0 *ρ* = −0.026; D15 *ρ* = 0.073; D30 *ρ* = 0.289) and PVAS (D0 *ρ* = 0.216; D15 *ρ* = −0.111; D30 *ρ* = −0.058).

**FIGURE 4:**
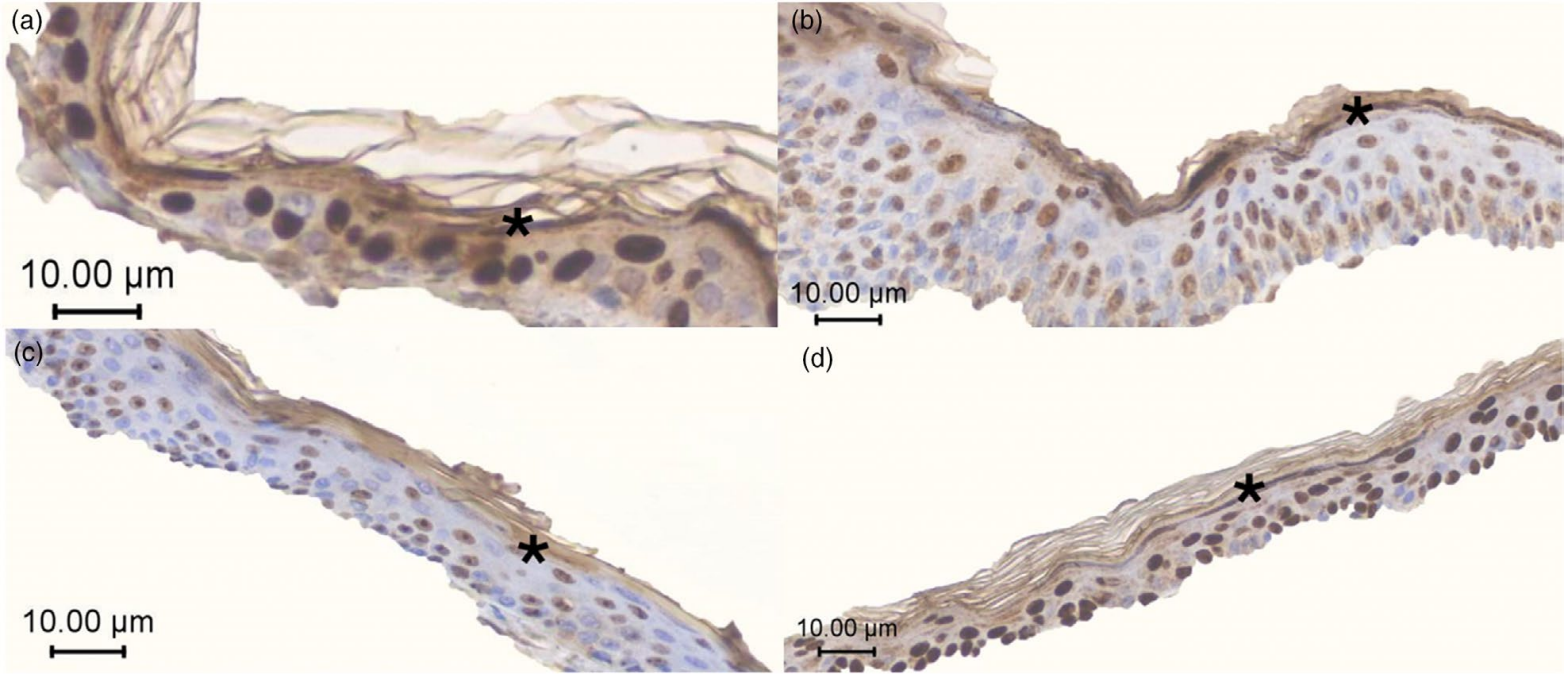
Representative filaggrin 2 immunolabelling in the stratum granulosum and stratum corneum by immunohistochemical investigation (asterisks). (a) Control skin; (b) atopic nonlesional skin on Day (D)0; (c) atopic lesional skin on D0 (erythema); and (d) D30 nonlesional skin (after oclacitinib maleate). ZEN blue edition (Zeiss) software.

Figure 4 shows representative images of FLG2 immunolabelling in skin sections from normal and atopic dogs (lesional and nonlesional) on D0 and atopic on D30 (nonlesional), after oclacitinib maleate administration.

## DISCUSSION

The results of this study showed significantly higher FLG2 immunostaining in control dogs than in atopic dogs (lesional and nonlesional), as described previously.^5^, ^6^, ^21^ Moreover, there was a significant increase in FLG2 expression on D30 nonlesional skin compared with atopic nonlesional skin on D0, suggesting a positive impact of oclacitinib maleate on the physical skin barrier, possibly linked to the blocking effect of the drug on Th2 cytokines. This suggests that cutaneous inflammation plays a key role in disrupting the skin barrier in dogs with AD. Another interesting finding was the nonsignificant difference between control and atopic skin on D30; thus, it can be speculated that the use of oclacitinib maleate could improve FLG2 expression in atopic dogs at a similar level to healthy skin. These findings support our hypothesis, specifically in nonlesional atopic skin.

Most humans with AD have altered FLG expression on either a genetic^23^ or acquired basis (e.g. Th2 inflammation and repetitive scratching).^16^ Alarmins, including IL‐33 and TSLP, and type 2 inflammatory mediators, such as IL‐4, IL‐13 and IL‐31, can reduce FLG expression. This has also been described in cutaneous inflammation mediated by Th17 (IL‐17), Th22 (IL‐22) and Th1 (IL‐1α, IL‐1β and tumour necrosis factor‐α). Likewise, other innate immune effector cells, including eosinophils, basophils and mast cells, increase the release of mediators that aggravate inflammation and worsen skin barrier disorders by downregulating stratum corneum structural proteins.^16^ Type 2 inflammatory cytokines and alarmins can also contribute to the itch–scratch cycle by activating pruritogenic neurons, intensifying skin barrier disruption and allowing bacterial colonisation, hence perpetuating the inflammatory responses.^16^

Prominent Th2‐polarised immune responses also have been demonstrated in cAD with variably increased levels of IL‐4, IL‐5 and IL‐13 in the serum, peripheral blood mononuclear cells and lesional skin, as well as increased numbers of Th2 cells in the peripheral circulation.^14^ However, it is unclear whether decreased FLG expression in atopic dogs is the result of a primary skin barrier defect (genetic) and/or secondary to inflammation.^21^ One study showed that treatment of canine reconstructed epidermis with Th2 cytokines decreases FLG immunodetection,^24^ suggesting an impact of inflammation on this protein. Other researchers found that treatment with ciclosporin led to an increase in FLG expression compared to that before treatment.^25^ Considering these findings, modulation or suppression of Th2 cytokines could be beneficial and secondarily improve skin barrier function^21^ as described in the present study with the use of oclacitinib maleate. This could represent additional evidence of the possible involvement of allergic inflammation as a main actor in cutaneous barrier defects in dogs.

Oclacitinib maleate acts as an inhibitor of the function of important pro‐inflammatory, pro‐allergic and pruritogenic cytokines via inhibition of the JAK signal transducer and activator of transcription signalling pathway.^26^ The suppressive effect of this drug on Th2 responses, leading to a reduction in skin inflammation, could improve FLG2 expression in nonlesional atopic skin in this study, after 4 weeks of its use. Conversely, this effect was not statistically significant on lesional skin, implying a promising proactive, not reactive, effect on cutaneous barrier structure, or possibly the need for longer treatments to improve FLG2 expression on the more modified lesional skin. This could be explained by the fact that IL‐31 is one of the earliest and most pivotal cytokines involved in acute cAD. One study showed upregulation of messenger RNA encoding IL‐31 in the skin of atopic dogs as early as 6 h after allergen challenge, when erythema was not yet evident.^27^ However, the same study demonstrated that 48 h after allergen challenge, there was amplification of additional Th2 (IL‐4, IL‐5, IL‐13, IL‐31 and IL‐33), Th9 (IL‐9) and Th22 (IL‐22) cytokines, as well as Th2‐promoting chemokines,^27^ some of them being pathways that escape from the oclacitinib maleate modeof action, which could justify the nonsignificant improvement of FLG2 expression in atopic dogs exhibiting erythema (lesional skin). Additionally, the description of these transcriptomes^8^ could explicate the lack of positive correlation between FLG2 expression and clinical scores (CADESI‐04 and PVAS) in this study. Exaggerated inflammatory responses may result in widely diffused secondary changes that influence the AD phenotype, such as suppression of epidermal differentiation genes, including FLG, by Th2, Th22 and Th1 cytokines,^28^ as well as excessive degradation of FLG and disorders in the processing of profilaggrin, as described in mice and humans.^29^

The primary custom‐made anti‐canine‐filaggrin polyclonal antibody used in this study was directed to canine FLG2. The *FLG2* gene has similarities in genomic sequence as well as protein distribution with *FLG*, and its participation in skin barrier defects has been proposed in humans and mice.^30^, ^31^ A comparison between the updated canine database and its human counterpart suggested that the previously identified canine *FLG* gene^5^, ^7^, ^8^, ^32^, ^33^, ^34^, ^35^ is more similar to human *FLG2* than to the human *FLG* gene. A recent study evaluated the expression of both filaggrins, FLG and FLG2, in normal and atopic skin biopsies from dogs before and after allergen challenges, by immunohistochemical investigation and Western blot. The authors concluded that staining using an antibody for canine FLG^36^ and one for canine FLG2 results in a comparable epidermal location, suggesting a similar distribution, and not a similar behaviour, of these proteins in canine skin.^12^ We observed that canine atopic skin has a significantly decreased expression of FLG2, compared to healthy skin. This finding reinforces the likelihood of FLG2 affectations in dogs with AD.

Limitations of this study include the small number of animals, the variability of breeds and the short period of oclacitinib maleate use assessed. However, the differences in FLG2 expression observed between healthy and atopic dogs appear to be similar in all animals, despite the different breeds included, leading to statistically significant results.

In conclusion, we observed a significant increase in FLG2 expression in atopic nonlesional skin after 30 days of oclacitinib maleate administration. These results suggest that this drug could have a positive impact on cutaneous barrier structure, improving FLG2 expression by decreasing inflammation and cutaneous trauma. This could represent an additional benefit of oclacitinib maleate as a proactive therapy, leading to a possible improvement of the skin barrier in dogs with subclinical or mild cutaneous inflammation. Further studies with more animals and more extended study periods are needed to understand the extent to which therapies targeting type 2 inflammatory responses can improve skin barrier function and how they can impact clinical manifestations in dogs with AD.

## AUTHOR CONTRIBUTIONS

**Wendie Roldan Villalobos:** Conceptualization; investigation; writing – original draft; methodology; validation; writing – review and editing; supervision. **Tássia Ferreira:** Investigation; methodology; validation. **Fernanda Borek:** Investigation; methodology; validation. **Domenico Santoro:** Investigation; methodology; validation. **Lluis Ferrer:** Investigation; validation; writing – review and editing; supervision. **Marconi Farias:** Conceptualization; investigation; methodology; validation; writing – review and editing; supervision.

## FUNDING INFORMATION

This study was self‐funded. W.R. received a PhD grant from the CAPES (Coordenação de aperfeiçoamento de pessoal de nível superior)—Brazil.

## CONFLICT OF INTEREST STATEMENT

No conflicts of interest have been declared.

## Supporting information


Figure S1

## Data Availability

The data that support the findings of this study are available from the corresponding author upon reasonable request.

## References

[1] Marsella R . Advances in our understanding of canine atopic dermatitis. Vet Dermatol. 2021;32:547–e151. 33891338 10.1111/vde.12965

[2] Segarra S , Naiken T , Garnier J , Hamon V , Coussay N , Bernard F‐X . Enhanced in vitro expression of filaggrin and antimicrobial peptides following application of glycosaminoglycans and a sphingomyelin‐rich lipid extract. Vet Sci. 2022;9:323. 35878340 10.3390/vetsci9070323 PMC9316723

[3] Zhu TH , Zhu TR , Tran KA , Sivamani RK , Shi VY . Epithelial barrier dysfunctions in atopic dermatitis: a skin–gut–lung model linking microbiome alteration and immune dysregulation. Br J Dermatol. 2018;179:570–581. 29761483 10.1111/bjd.16734

[4] Combarros D , Cadiergues M‐C , Simon M . Update on canine filaggrin: a review. Vet Q. 2020;40:162–168. 32308144 10.1080/01652176.2020.1758357 PMC7241532

[5] Chervet L , Galichet A , McLean WHI , Chen H , Suter MM , Roosje PJ , et al. Missing C‐terminal filaggrin expression, NFkappaB activation and hyperproliferation identify the dog as a putative model to study epidermal dysfunction in atopic dermatitis. Exp Dermatol. 2010;19:e343–e346. 20626465 10.1111/j.1600-0625.2010.01109.x

[6] Marsella R , Samuelson D , Harrington L . Immunohistochemical evaluation of filaggrin polyclonal antibody in atopic and normal beagles. Vet Dermatol. 2009;20:547–554. 20178493 10.1111/j.1365-3164.2009.00844.x

[7] Roque JB , O'Leary CA , Kyaw‐Tanner M , Duffy DL , Shipstone M . Real‐time PCR quantification of the canine filaggrin orthologue in the skin of atopic and non‐atopic dogs: a pilot study. BMC Res Notes. 2011;4:554. 22188733 10.1186/1756-0500-4-554 PMC3339370

[8] Theerawatanasirikul S , Sailasuta A , Thanawongnuwech R , Suriyaphol G . Alterations of keratins, involucrin and filaggrin gene expression in canine atopic dermatitis. Res Vet Sci. 2012;93:1287–1292. 22784629 10.1016/j.rvsc.2012.06.005

[9] Makino T , Mizawa M , Yamakoshi T , Takaishi M , Shimizu T . Expression of filaggrin‐2 protein in the epidermis of human skin diseases: a comparative analysis with filaggrin. Biochem Biophys Res Commun. 2014;449:100–106. 24813994 10.1016/j.bbrc.2014.04.165

[10] Donovan M , Salamito M , Thomas‐Collignon A , Simonetti L , Desbouis S , Rain J‐C , et al. Filaggrin and filaggrin 2 processing are linked together through skin aspartic acid protease activation. PLoS One. 2020;15:e0232679. 32437351 10.1371/journal.pone.0232679 PMC7241785

[11] Santoro D , Marsella R , Ahrens K , Graves TK , Bunick D . Altered mRNA and protein expression of filaggrin in the skin of a canine animal model for atopic dermatitis. Vet Dermatol. 2013;24:329–336. 23668858 10.1111/vde.12031

[12] Marsella R , Ahrens K , Wilkes R . Studies using antibodies against filaggrin and filaggrin 2 in canine normal and atopic skin biopsies. Animals (Basel). 2024;14:478. 38338121 10.3390/ani14030478 PMC10854974

[13] Auriemma M , Vianale G , Amerio P , Reale M . Cytokines and T cells in atopic dermatitis. Eur Cytokine Netw. 2013;24:37–44. 23608610 10.1684/ecn.2013.0333

[14] Früh SP , Saikia M , Eule J , Mazulis CA , Miller JE , Cowulich JM , et al. Elevated circulating Th2 but not group 2 innate lymphoid cell responses characterize canine atopic dermatitis. Vet Immunol Immunopathol. 2020;221:110015. 32058160 10.1016/j.vetimm.2020.110015

[15] Furue M , Furue M . Interleukin‐31 and pruritic skin. J Clin Med. 2021;10:1906. 33924978 10.3390/jcm10091906 PMC8124688

[16] Beck LA , Cork MJ , Amagai M , De Benedetto A , Kabashima K , Hamilton JD , et al. Type 2 inflammation contributes to skin barrier dysfunction in atopic dermatitis. JID Innov. 2022;2:100131. 36059592 10.1016/j.xjidi.2022.100131 PMC9428921

[17] Gonzales AJ , Bowman JW , Fici GJ , Zhang M , Mann DW , Mitton‐Fry M . Oclacitinib (APOQUEL®) is a novel Janus kinase inhibitor with activity against cytokines involved in allergy. J Vet Pharmacol Ther. 2014;37:317–324. 24495176 10.1111/jvp.12101 PMC4265276

[18] Favrot C , Steffan J , Seewald W , Picco F . A prospective study on the clinical features of chronic canine atopic dermatitis and its diagnosis. Vet Dermatol. 2010;21:23–31. 20187911 10.1111/j.1365-3164.2009.00758.x

[19] Olivry T , Saridomichelakis M , Nuttall T , Bensignor E , Griffin CE , Hill PB , et al. Validation of the canine atopic dermatitis extent and severity index (CADESI)‐4, a simplified severity scale for assessing skin lesions of atopic dermatitis in dogs. Vet Dermatol. 2014;25:77–85. 24461108 10.1111/vde.12107

[20] Rybníček J , Lau‐Gillard PJ , Harvey R , Hill PB . Further validation of a pruritus severity scale for use in dogs. Vet Dermatol. 2009;20:115–122. 19171021 10.1111/j.1365-3164.2008.00728.x

[21] Marsella R , Santoro D , Ahrens K , Thomas AL . Investigation of the effect of probiotic exposure on filaggrin expression in an experimental model of canine atopic dermatitis. Vet Dermatol. 2013;24:260 e57. 23432387 10.1111/vde.12006

[22] Marsella R . Does filaggrin expression correlate with severity of clinical signs in dogs with atopic dermatitis? Vet Dermatol. 2013;24:266 e59. 23398596 10.1111/vde.12007

[23] Irvine AD , Mclean WHI , Leung DYM . Filaggrin mutations associated with skin and allergic diseases. N Engl J Med. 2011;365:1315–1327. 21991953 10.1056/NEJMra1011040

[24] Pin D , Pendaries V , Keita Alassane S , Froment C , Amalric N , Cadiergues M‐C , et al. Refined immunochemical characterization in healthy dog skin of the epidermal cornification proteins, filaggrin, and corneodesmosin. J Histochem Cytochem. 2019;67:85–97. 30199656 10.1369/0022155418798807 PMC6354317

[25] White AG , Santoro D , Ahrens K , Marsella R . Single blinded, randomized, placebo‐controlled study on the effects of ciclosporin on cutaneous barrier function and immunological response in atopic beagles. Vet Immunol Immunopathol. 2018;197:93–101. 29475513 10.1016/j.vetimm.2018.02.001

[26] Jasiecka‐Mikołajczyk A , Jaroszewski JJ , Maślanka T . Oclacitinib, a janus kinase inhibitor, reduces the frequency of IL‐4‐and IL‐10‐, but not IFN‐γ‐, producing murine CD4+ and CD8+ T cells and counteracts the induction of type 1 regulatory T cells. Molecules. 2021;26:5655. 34577127 10.3390/molecules26185655 PMC8472008

[27] Olivry T , Mayhew D , Paps JS , Linder KE , Peredo C , Rajpal D , et al. Early activation of Th2/Th22 inflammatory and pruritogenic pathways in acute canine atopic dermatitis skin lesions. J Invest Dermatol. 2016;136:1961–1969. 27342734 10.1016/j.jid.2016.05.117

[28] Tsakok T , Woolf R , Smith CH , Weidinger S , Flohr C . Atopic dermatitis: the skin barrier and beyond. Br J Dermatol. 2019;180:464–474. 29969827 10.1111/bjd.16934

[29] Bonnart C , Deraison C , Lacroix M , Uchida Y , Besson C , Robin A , et al. Elastase 2 is expressed in human and mouse epidermis and impairs skin barrier function in Netherton syndrome through filaggrin and lipid misprocessing. J Clin Invest. 2010;120:871–882. 20179351 10.1172/JCI41440 PMC2827963

[30] Wu Z , Hansmann B , Meyer‐Hoffert U , Gläser R , Schröder J‐M . Molecular identification and expression analysis of filaggrin‐2, a member of the S100 fused‐type protein family. PLoS One. 2009;4:e5227. 19384417 10.1371/journal.pone.0005227 PMC2668185

[31] Hansmann B , Ahrens K , Wu Z , Proksch E , Meyer‐Hoffert U , Schröder J‐M . Murine filaggrin‐2 is involved in epithelial barrier function and down‐regulated in metabolically induced skin barrier dysfunction. Exp Dermatol. 2012;21:271–276. 22417302 10.1111/j.1600-0625.2012.01449.x

[32] Wood SH , Ollier WE , Nuttall T , McEwan NA , Carter SD . Despite identifying some shared gene associations with human atopic dermatitis the use of multiple dog breeds from various locations limits detection of gene associations in canine atopic dermatitis. Vet Immunol Immunopathol. 2010;138:193–197. 20728225 10.1016/j.vetimm.2010.07.020

[33] Suriyaphol G , Suriyaphol P , Sarikaputi M , Theerawatanasirikul S , Sailasuta A . Association of filaggrin (*FLG*) gene polymorphism with canine atopic dermatitis in small breed dogs. Thai J Vet Med. 2011;41:509–517.

[34] Barros Roque J , O'Leary CA , Kyaw‐Tanner M , Latter M , Mason K , Shipstone M , et al. Haplotype sharing excludes canine orthologous filaggrin locus in atopy in West Highland white terriers. Anim Genet. 2009;40:793–794. 19466940 10.1111/j.1365-2052.2009.01915.x

[35] Agler CS , Friedenberg S , Olivry T , Meurs KM , Olby NJ . Genome‐wide association analysis in West Highland white terriers with atopic dermatitis. Vet Immunol Immunopathol. 2019;209:1–6. 30885300 10.1016/j.vetimm.2019.01.004

[36] Kanda S , Sasaki T , Shiohama A , Nishifuji K , Amagai M , Iwasaki T , et al. Characterization of canine filaggrin: gene structure and protein expression in dog skin. Vet Dermatol. 2013;24:25–31, e7. 23331676 10.1111/j.1365-3164.2012.01105.x

