# Supplementary material for: Evaluation of filaggrin 2 expression in dogs with atopic dermatitis before and after oclacitinib maleate administration
Source: Vet Dermatol. 2025 Mar 5;36(4):453–61. doi: 10.1111/vde.13334 (PMC12243448; doi:10.1111/vde.13334)
Supplement: Supplementary file 1 — Figure S1 [file VDE-36-453-s001.docx]

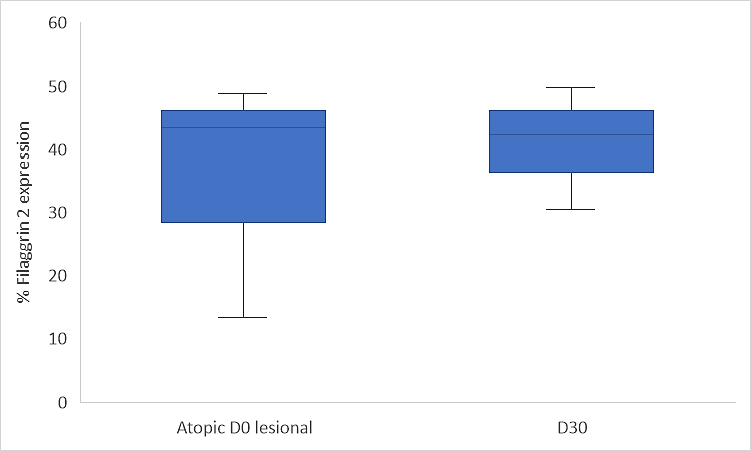


*p*=0.688

Comparison of filaggrin expression in lesional atopic skin on D0 (before oclacitinib maleate) and D30 non-lesional (after oclacitinib maleate).
